# The Prevention Role of Theaflavin-3,3′-digallate in Angiotensin II Induced Pathological Cardiac Hypertrophy via CaN-NFAT Signal Pathway

**DOI:** 10.3390/nu14071391

**Published:** 2022-03-26

**Authors:** Hui Zhou, Chen Xia, Yaqing Yang, Hasitha Kalhari Warusawitharana, Xiaohui Liu, Youying Tu

**Affiliations:** 1 Department of Tea Science, Zhejiang University, Hangzhou 310058, China; 3150102861@zju.edu.cn (H.Z.); lukexia@tealab.cn (C.X.); 21916132@zju.edn.cn (Y.Y.); 11816127@zju.edu.cn (H.K.W.); 2 College of Tea Science, Yunnan Agricultural University, Kunming 650201, China

**Keywords:** theaflavin-3,3′-digallate, pathological cardiac hypertrophy, oxidative stress, calcium ion, calcineurin, nuclear factor of activated T cells

## Abstract

Theaflavin-3,3′-digallate (TF3) is a representative theaflavin of black tea and is remarkable for the anti-coronary heart disease effect. As an adaptive response to heart failure, pathological cardiac hypertrophy (PCH) has attracted great interest. In this study, the PCH cell model was established with H9c2 cells by angiotensin II, and the prevention effect and mechanisms of TF3 were investigated. The results showed that the cell size and fetal gene mRNA level were significantly reduced as pretreated with TF3 at the concentration range of 1–10 μM, also the balance of the redox system was recovered by TF3 at the concentration of 10 μM. The intracellular Ca^2+^ level decreased, Calcineurin (CaN) expression was down-regulated and the p-NFATc3 expression was up-regulated. These results indicated that TF3 could inhibit the activation of the CaN-NFAT signal pathway to prevent PCH, and TF3 may be a potentially effective natural compound for PCH and heart failure.

## 1. Introduction

Heart failure (HF) is a kind of heart circulation disorder syndrome characterized by impairment of cardiac function, venous blood stasis, and insufficient blood perfusion of the arterial system, affecting about 26 million people worldwide [1]. Pathological cardiac hypertrophy (PCH) is a kind of cardiovascular disease induced by pressure stimulation such as hypertension, during which the cardiomyocytes become slender, lose contractile function, and undergo oxidative stress. Since PCH is an early manifestation of HF [2], preventing PCH is of great scientific importance for the prevalence of HF. Nitrendipine, a clinical drug reported with the ability to prevent PCH in rats, is also accompanied by side effects, such as headache, dizziness, and palpitation [3]. Therefore, searching for potential drugs with safety and efficiency is urgent in this field.

Tea (*Camellia sinensis* (L.) O. Kuntze) is one of the most consumed beverages in the world. The anti-cardiovascular effect is widely studied. A prospective epidemiological study of 76,979 Japanese revealed that consuming more than one cup of green tea or oolong tea per day significantly reduced the risk of coronary heart disease [4]. Another follow-up survey containing 37,514 participants demonstrated that black tea consumption was negatively correlated with the prevalence of coronary heart disease [5]. The health benefit was recognized as being associated with tea polyphenols and theaflavins, which are reported to possess excellent antioxidant and free radical scavenging ability [6,7,8].

In recent years, tea bioactive substances with the ability to prevent PCH induced by angiotensin II (ANGII) or phenylephrine were developed in a series of literature. Epigallocatechin gallate (EGCG) was found to prevent phenylephrine-induced PCH by activating the AMPK pathway [9]. Kaempferol could inhibit the activity of the ASK1/JNK1/2/p38 signaling pathway in PCH cells [10]. The mechanisms were also focused on the rebalancing of the redox system. EGCG and Kaempferol blocked the production of ROS [10,11]. Gallic acid down-regulated the mRNA level of Nox2, a kind of oxidase [12].

Tea pigments account for approximately 20% of substances in black tea infusion, including theaflavins, thearubigins, and theabrownines. As an oxidation product of catechins and a characteristic compound of black tea, theaflavin-3,3’-digallate (TF3) has been reported to have the ability to reduce endoplasmic reticulum stress and protect the cardiovascular system [13]. However, the effects of TF3 in preventing PCH are never reported. In this research, the H9c2 cell line, derived from rat embryonic heart tissue was chosen to establish a PCH cell model in vitro with ANGII [14], the molecular mechanism of TF3 on hypertrophic signal transduction was elucidated.

## 2. Materials and Methods

### 2.1. Materials

Rat cardiomyoblast H9c2 cell line was purchased from the National Collection of Authenticated Cell Cultures (Shanghai, China). TF3 (92.4% purity) was obtained according to our lab’s previous method [15]. Antibodies against CaN, phospho Ser165 NFATc3, and glyceraldehyde-3-phosphate dehydrogenase (GAPDH) were purchased from Abcam (Cambridge, England). Fluorescent indicator Fluo-4 AM and Western blot analysis-related reagents were purchased from Beyotime Biotechnology (Shanghai, China). Other reagents used in this experiment were purchased from Meilunbio (Dalian, China).

### 2.2. Cell Culture and Treatments

H9c2 cells were cultured in high glucose Dulbecco’s Modified Eagle’s Medium (DMEM), supplemented with 10% fetal bovine serum (FBS), 100 U/mL penicillin, 100 μg/mL streptomycin, 584 mg/L L-glutamine, and 110 mg/L sodium pyruvate in an incubator (37 °C, 5% CO_2_). The PCH cell model was produced by adding 0.5 μM ANGII and incubated for 24 h. Cells were divided into 6 groups: Control group; Model group (0.5 μM ANGII induction). TFL, TFM, TFH treatment group (1, 5, 10 μM TF3 treatment); EGCG treatment group (50 μM EGCG treatment). All treatment groups began to add ANGII after 1 h of TF3 or EGCG treatment.

### 2.3. Cell Viability Determination

Cell Counting Kit-8 (CCK-8) was applied to determine cell viability. Cells were treated with different concentrations of TF3 for 1 h or ANGII for 24 h. After incubation, the cell culture medium was discarded and 100 μL of 10% CCK-8 solution was added to each well and incubated for 1 h, the absorbance values of each well were obtained at 450 nm using an ELISA microplate reader (Thermo, Multiscan MK3, Waltham, MA, USA).

### 2.4. F-actin and Nucleus Staining

After ANGII induction, cells were fixed in 4% paraformaldehyde for 10 min, stimulated by 0.1% TritonX-100 for 5 min, and stained with TRITC Phalloidin solution (1 μg/mL) and 4′,6-diamidino-2-phenylindole dihydrochloride (DAPI, 10 μg/mL) for 1 h. Cells were rinsed with phosphate buffer solution (PBS) three times between every two steps above. The images of F-actin and nucleus were photographed by a laser scanning confocal microscope (Olympus Corporation, Tokyo, Japan). ImageJ software (vision 1.53c, National Institutes of Health, Bethesda, MD, USA) was applied to analyze cell size. At least 50 cells were measured in each group).

### 2.5. Total RNA Extraction and Real-Time PCR Analysis for Fetal Genes

Total RNA in each group was extracted by the Eastep® Super Total RNA Extraction Kit (Promega, Madison, WI, USA). RNA quantity and purity were determined using a NanoDrop spectrophotometer (ThermoFisher Scientific, Waltham, MA, USA). RNA from each sample (800 ng) was reverse transcribed into cDNA using the PrimeScript™ RT reagent Kit with gDNA Eraser (Takara, Shiga, Japan). cDNA from each group underwent quantitative real-time PCR using TB Green® *Premix Ex Taq*™ II (Takara, Shiga, Japan). The sequences of the primers are presented in Table 1. GAPDH was considered a reference gene and the expression ratio of a target gene versus a reference gene was determined according to the 2^−ΔΔCt^ method.

### 2.6. Western Blot Analysis

Ice-cold RIPA lysis solution (containing 1% Triton X-100, 1% deoxycholate, 0.1% SDS, 1 mM PMSF and 1% phosphatase inhibitor) was used to extract total protein from cells. A BCA protein assay kit was used to determine protein concentration. After quantification, all samples were diluted into equal concentrations.

Western blot analysis was carried out according to Gao with modifications [16]. In brief, proteins from each group were separated by SDS-PAGE gel electrophoresis and transferred to PVDF membranes. After blocking for 1 h, membranes were incubated overnight at 4 °C with corresponding primary antibodies (CaN, p-NFATc3, and GAPDH). Then, membranes were washed three times with ultrapure water and immersed in the secondary antibody solution containing goat anti-rabbit IgG(H+L) for 2 h. After washing, liquid A and liquid B of the ECL kit were mixed and added to the membrane surface. Images were photographed using a fluorescence chemiluminescence imaging system (Fujifilm, Tokyo, Japan). ImageJ was used to analyze the optical density of each gel lane. Target protein expression levels were normalized to the GAPDH protein.

### 2.7. Reactive Oxygen Species Level Determination

Fluorescent indicator 2′,7′-dichlorodihydrofluorescein diacetate (DCFH-DA) was applied to quantify intracellular reactive oxygen species (ROS) level. After ANGII induction, cells were rinsed three times with PBS and incubated with DCFH-DA (10 μM) for 20 min. The DCFH fluorescent intensity was measured using a fluorescence microplate reader (Schuett-Biotec, Germany), according to the manufacturer’s instructions.

### 2.8. Superoxide Dismutase Activity, Catalase Activity, and Nitric Oxide Level Determination

Cell lysates were centrifuged at 14,000× *g* at 4 °C for 5 min. The supernatants were transferred to new microtubes. Superoxide dismutase (SOD) assay kit, catalase (CAT) assay kit, and nitric oxide (NO) assay kit (Nanjing Jiancheng Bioengineering Institute, Nanjing, China) were used to determine the SOD activity, CAT activity, and NO level, respectively, according to the manufacturer’s instructions. Briefly, samples of each group were added into tubes or microplates to mix with reagents in kits. After reaction for the required time, the absorption of each tube or microplate was read using an ELISA microplate reader. Finally, the NO level, SOD, and CAT activities were calculated. The data were quantified by corresponding protein content.

### 2.9. Intracellular Ca^2+^ Level Determination

Fluorescent indicator Fluo-4 AM was used to quantify intracellular Ca^2+^ level. After ANGII induction, cells were rinsed three times with PBS and then incubated with Fluo-4 AM (5 μM) for 30 min. The incubated cells were rinsed with PBS and then incubated for another 20 min. The Fluo-4 fluorescent intensity was measured by a fluorescence microplate reader, according to the manufacturer’s instructions.

### 2.10. Statistical Analysis

The data were expressed as the mean ± standard deviation (SD) from three replicates. All results were analyzed by one-way analysis of variance (ANOVA) with Statistical Analysis System (SAS, SAS Institute Inc., Cary, NC, USA). Student–Newman–Keuls (SNK) multiple-range test was applied to reveal the differences between treatments with a significance level of 0.05 and an extremely significant level of 0.01.

## 3. Results

### 3.1. H9c2 Cell PCH Model Induction

**Figure 1:**
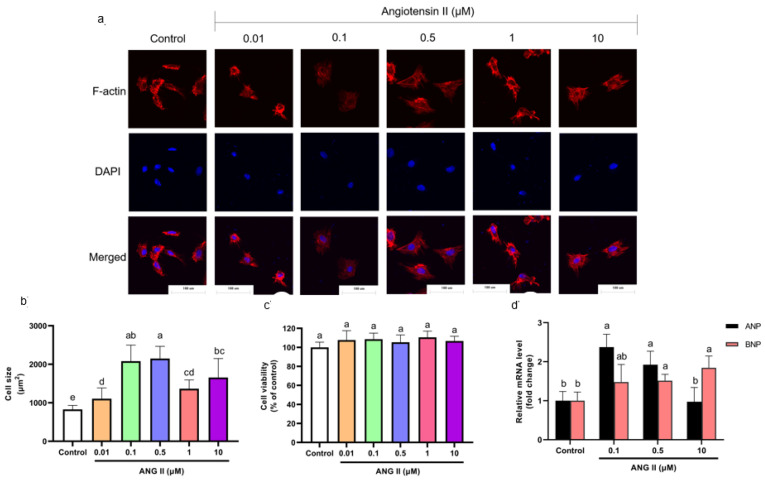
(**a**) Morphology change of H9c2 cells treated with different concentrations of ANGII (×400). (**b**) Size of H9c2 cells treated with ANGII. (**c**) Viability of H9c2 cells treated with ANGII. (**d**) ANP and BNP mRNA level of H9c2 cells treated with ANGII. Significant differences between different treatments were shown by different letters (*p* < 0.05).

Cells were incubated with different concentrations of ANGII (0.01, 0.1, 0.5, 1, and 10 μM) for 24 h to select an appropriate concentration of ANGII for this experiment. The H9c2 cell PCH model is characterized by a significant size increase and fetal gene expression [17,18]. According to F-actin staining results, cell size increased significantly as the concentration of ANGII ranged from 0.1–10 μM (Figure 1a,b). The cytotoxicity of ANGII on H9c2 cells was analyzed using a CCK-8 assay. As shown in Figure 1c, there was no significant difference in cell viability between ANGII treatments (0.1–10 μM) and the control group; the result indicated that ANGII treatment had no inhibition on cell proliferation. In Figure 1d, different concentrations of ANGII had distinct effects on ANP and BNP mRNA levels. Both ANP and BNP mRNA levels increased only when the ANGII concentration was 0.5 μM (92.38% and 51.47%, respectively), which suggested PCH cells appeared. Based on these results, it was confirmed that the appropriate concentration for the ANGII-induced cell model was 0.5 μM.

### 3.2. Effect of TF3 on Cell Viability

**Figure 2:**
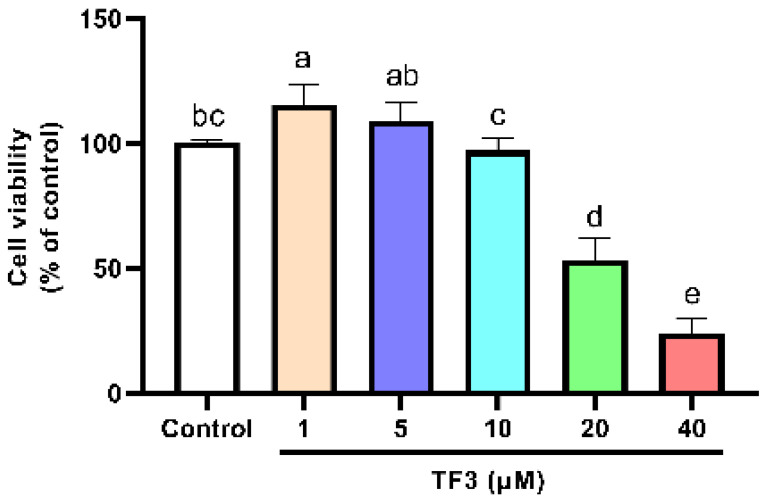
Viability of H9c2 cells treated with different concentrations of TF3. Significant differences between different treatments were shown by different letters (*p* < 0.05).

The cell viability of TF3 treatment groups was also determined. The cell viability significantly decreased as the concentration of TF3 was higher than 10 μM (Figure 2). For the sake of developing anti-hypertrophy compounds with low risk, 1, 5, and 10 μM of TF3 were chosen for the following experiments. EGCG 50 μM was set as the positive control. It showed no inhibition effect on cell viability (Figure S1).

### 3.3. TF3 Pretreatment Alleviated Cell Hypertrophy and Fetal Gene Expression

**Figure 3:**
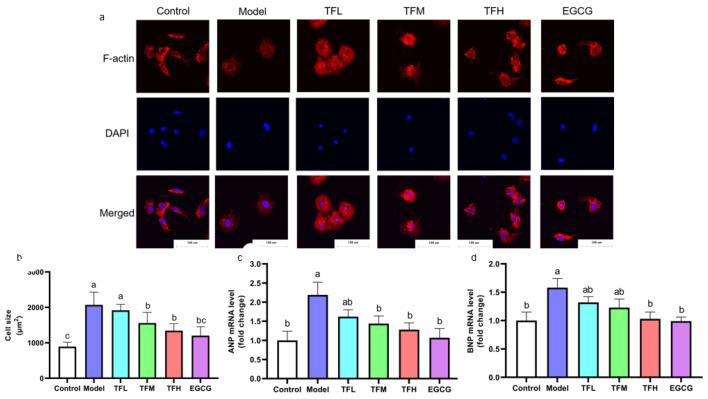
(**a**) Morphology change of H9c2 cells treated with different concentrations of TF3 and EGCG (×400). (**b**) Size of H9c2 cells treated with TF3 and EGCG. (**c**) ANP mRNA level of H9c2 cells treated with TF3 and EGCG. (**d**) BNP mRNA level of H9c2 cells treated with TF3 and EGCG. Control group, no ANGII treatment; Model group, ANGII 0.5 μM induction; TFL group, TF3 1 μM pretreatment and ANGII 0.5 μM induction; TFM group, TF3 5 μM pretreatment and ANGII 0.5 μM induction; TFH group, TF3 10 μM pretreatment and ANGII 0.5 μM induction; EGCG group, EGCG 50 μM pretreatment and ANGII 0.5 μM induction. Significant differences between different treatments were shown by different letters (*p* < 0.05).

To demonstrate the preventing effect of TF3 on PCH, cell size and fetal gene mRNA levels were determined. TF3 pretreatment alleviated the cell size increase compared to the control group (13.0–73.8%), as shown in Figure 3. Cell morphology changed from spindle to round in the Model group. TFM, TFH, and EGCG treatment groups significantly prevented cell hypertrophy and impeded cell morphological changes (*p* < 0.01). ANP and BNP mRNA levels increased after ANGII stimulation and were remarkably constrained when pretreated with TF3 (47.9–76.5% and 44.7–94.8%, Figure 3c,d). In conclusion, different concentrations of TF3 could prevent PCH. The TFH group showed a similar anti-hypertrophic effect to EGCG.

### 3.4. TF3 Pretreatment Rebalance of Redox System in PCH Model

**Figure 4:**
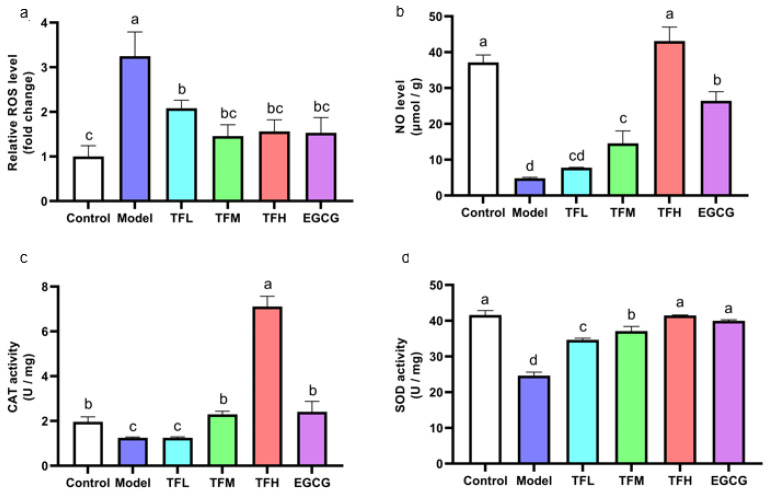
(**a**) ROS level of H9c2 cells treated with different concentrations of TF3 and EGCG. (**b**) NO level of H9c2 cells treated with TF3 and EGCG. (**c**) CAT activity of H9c2 cells treated with TF3 and EGCG. (**d**) SOD activity of H9c2 cells treated with TF3 and EGCG. Control group, no ANGII treatment; Model group, ANGII 0.5 μM induction; TFL group, TF3 1 μM pretreatment and ANGII 0.5 μM induction; TFM group, TF3 5 μM pretreatment and ANGII 0.5 μM induction; TFH group, TF3 10 μM pretreatment and ANGII 0.5 μM induction; EGCG group, EGCG 50 μM pretreatment and ANGII 0.5 μM induction. Significant differences between different treatments were shown by different letters (*p* < 0.05).

As the cellular oxidation stress could be reflected by the ROS level, the rebalance of redox system equilibrium could be concluded by comparison between the Model group and treatment group. DCFH fluorescence density results showed that ANGII stimulation significantly increased the intracellular ROS level (approximately 3.2 times of the Control group). However, TF3 1–10 μM significantly decreased the ROS level (*p* < 0.01, Figure 4a). Among all groups, TFM presented the best inhibition effect (79.6%).

In Figure 4b, the NO level in PCH cells was remarkably inhibited (approximately 12.9% of the Control group). Pretreatment of TF3 5 or 10 μM significantly prevented the decrease in NO (*p* < 0.01). The NO level in the TFH group was similar to the Control group (*p* > 0.05).

In PCH model cells, CAT activity decreased significantly (*p* < 0.01, Figure 4c). TFM treatment recovered CAT activity to the normal level and TF3 10 μM could increase CAT activity to 3.6 times that of the Control group. SOD activity in the Model group was decreased to 59.2%. Pretreatment with TF3 regulated SOD activity (59.0–99.3%) and the effect of the TFH group was better than that of the EGCG group (Figure 4d).

In this experiment, ANGII stimulation increased the ROS level, reduced the NO level, and partially inactivated CAT and SOD activity. TF3 could recover the balance of the redox system and the effect of TF3 10 μM was better than EGCG 50 μM.

### 3.5. TF3 Pretreatment Inhibited the Increase of Ca^2+^ Level in the PCH Model

**Figure 5:**
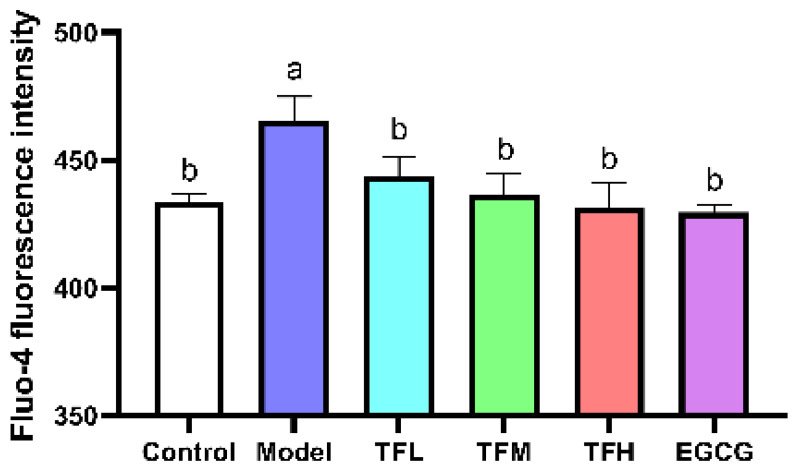
Fluo-4 fluorescence intensity of H9c2 cells treated with different concentrations of TF3 and EGCG. Control group, no ANGII treatment; Model group, ANGII 0.5 μM induction; TFL group, TF3 1 μM pretreatment and ANGII 0.5 μM induction; TFM group, TF3 5 μM pretreatment and ANGII 0.5 μM induction; TFH group, TF3 10 μM pretreatment and ANGII 0.5 μM induction; EGCG group, EGCG 50 μM pretreatment and ANGII 0.5 μM induction. Significant differences between different treatments were shown by different letters (*p* < 0.05).

Fluo-4 fluorescence intensity represented the intracellular Ca^2+^ level. In Figure 5, the fluorescence intensity was significantly enhanced after ANGII stimulation (*p* < 0.01), which meant intracellular Ca^2+^ accumulated and Ca^2+^-related pathways might be activated. TF3 treatment could reduce ANGII stimulation and regulate the Ca^2+^ level to a normal level (*p* > 0.05). This result suggested that TF3 may block the subsequent Ca^2+^-related pathways through controlling Ca^2+^ levels and prevent PCH eventually.

### 3.6. TF3 Pretreatment Prevented PCH through the CaN-NFAT Signal Pathway

**Figure 6:**
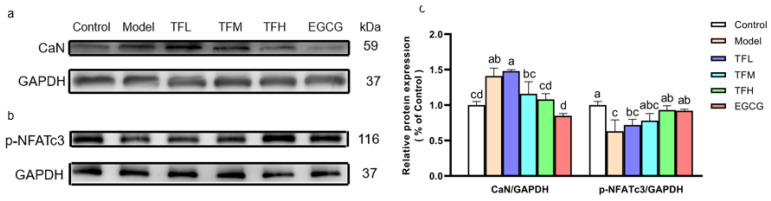
(**a**) CaN protein expression of H9c2 cells treated with different concentrations of TF3 and EGCG. (**b**) p-NFATc3 protein expression of H9c2 cells treated with TF3 and EGCG. (**c**) Densitometry analysis of protein bands. Control group, no ANGII treatment; Model group, ANGII 0.5 μM induction; TFL group, TF3 1μM pretreatment and ANGII 0.5 μM induction; TFM group, TF3 5 μM pretreatment and ANGII 0.5 μM induction; TFH group, TF3 10 μM pretreatment and ANGII 0.5 μM induction; EGCG group, EGCG 50 μM pretreatment and ANGII 0.5 μM induction. Significant differences between different treatments were shown by different letters (*p* < 0.05).

In Figure 6, Western blot results demonstrated that ANGII significantly increased the CaN protein level and meanwhile reduced the phosphorylation of NFATc3 at Ser165 (Figure 6a,b), which meant the CaN-NFAT signal pathway was activated in PCH cells. TF3 10 μM could remarkably decrease CaN expression (80.5%, *p* < 0.01) and reverse the change of the phosphorylation level of NFATc3 (81.1%, *p* < 0.01). The CaN and p-NFATc3 levels in the TFH group and the EGCG group had no significant difference from the Control group (Figure 6c). 

These results suggested that TF3 might decrease the ANP and BNP mRNA levels by inhibiting the activation of the CaN-NFAT signal pathway in PCH cells.

## 4. Discussion

Angiotensin substances are a series of peptides that can strongly constrict blood vessels and stimulate aldosterone secretion. ANGII is a universal hypertrophy promoter and induces PCH via the renin–angiotensin–aldosterone system. For example, C57BL/6 rats’ cardiomyocytes size significantly enlarged after chronic subcutaneous injection with 1 μg/kg/min of ANGII for 14 days [19]. ANP and BNP are considered to inhibit the effect of ANGII and are over-expressed in PCH cells. The expression of these genes can be used as indicators for PCH. In this study, ANGII was chosen to establish the PCH model, ANP and BNP mRNA levels increased significantly after 0.5 μM ANGII stimulation for 24 h. This may be caused by the inhibition of transcription and expression of natriuretic peptide receptor A (ANP and BNP receptors) [20]. In addition to ANP and BNP, the low-expression of the α-myosin heavy chain (α-MHC) and over-expression of the β-myosin heavy chain (β-MHC) gene expression were also observed in the PCH model [21,22].

Natural products have become potential targets because of their abundant source and safety advantages. Polyphenols have attracted extensive attention for their strong antioxidant capacities and free radical scavenging effects. Resveratrol, wogonin, gallic acid, kaempferol, and EGCG all showed protective effects against PCH [9,10,11,12,18,23]. Common structural features of these ingredients were phenolic hydroxyl groups, which may be related to PCH-related protein expression. Theaflavins are a series of pigments composed by the enzymatic oxidation of catechins. The number of phenolic hydroxyl groups in theaflavins is approximately twice that of catechins. TF3 is the highest content of theaflavin monomer in black tea. In this study, TF3 was selected as a potential anti-hypertrophy compound. EGCG 50 μM was picked as a positive control because it could prevent PCH [9]. The results showed that TF3 1–10 μM could significantly prevent PCH caused by ANGII. The effect of TF3 10 μM was comparable to EGCG 50 μM, which meant TF3 had a stronger prevention effect than EGCG. Besides, an extracellular solution can affect cell viability and life span through osmotic pressure. TF3 is regarded as a safer potential drug than EGCG.

Apart from ANGII, there are other neurohumoral factors that can cause PCH, for example, phenylephrine, isoproterenol, and vasopressin. Phenylephrine could induce PCH by inactivating the AMPK pathway and causing oxidative stress [9,10]. Isoproterenol could promote Akt and CREB phosphorylation and Swietenine, extracted from Swietenia macrophylla seed, prevented PCH caused by isoproterenol [24]. Vasopressin activated the MAPK signaling pathway and up-regulated CaN expression, thus inducing PCH [25]. In the above cases, some natural products (EGCG, Kaempferol, Swietenine) showed the preventative effect of PCH. As a characteristic component of tea, TF3 can be verified whether it has the effect of preventing PCH caused by phenylephrine, isoproterenol, and vasopressin in the future.

It is well-known that PCH is not an independent process. The occurrence of PCH is often accompanied by oxidative stress, inflammation, apoptosis, autophagy, cardiac fibrosis, and energy metabolism compensation [20,25,26,27,28,29]. ROS are a series of single-electron reduction products of oxygen produced by the mitochondria. Under normal circumstances, the intracellular redox system is in balance, and ROS are maintained at a specific level. The redox system balances dysfunctions in PCH cells, which directly leads to the increase in intracellular ROS levels [11,30]. CAT and SOD are two important antioxidant enzymes. In this study, ROS levels increased significantly after ANGII stimulation, and the activities of SOD and CAT decreased significantly. This might be due to the abnormal expression of fetal genes. Excessive fetal genes protein synthesis consumes vast energy. Mitochondria also produce excessive ROS when providing energy [31]. Meanwhile, antioxidant enzymes SOD and CAT were inactivated due to disulfide bonds formation of cysteine residues, which eventually leads to redox imbalance. However, different concentrations of TF3 or EGCG were found to eliminate oxidative stress caused by ANGII. SOD and CAT activities were related to the formation of H_2_O_2_. It could be reasonably inferred that TF3 has a good scavenging effect against H_2_O_2_, which has been confirmed [6]. NO is produced by arginine through the catalysis of nitric oxide synthase (NOS) and can dilate blood vessels. O_2_·^−^, a kind of ROS, can react with NO, which proved that NO is one of the signal molecules of the cell redox balance. In this study, the NO level declined in the Model group and TF3 could regulate NO production, which is consistent with previous research [32]. TF3 may have the same ability to promote eNOS activity as TF1 [33].

As concluded, TF3 could rebalance the redox system by scavenging ROS, recovering NO, and activating two antioxidant enzymes. It was also necessary to evaluate the effect of TF3 on oxidases and MDA in the future.

There are three main pathways related to PCH, namely the CaN-NFAT pathway, AMPK pathway, and MAPK pathway. It has been shown that the activation of the AMPK pathway inhibits the development of PCH via a number of sub-pathways, such as eukaryotic elongation factor-2 (eEF2), p70S6 kinase (p70S6K), and mammalian target of rapamycin (mTOR) pathway [34,35]. Hyperactivity of MAPKs (JNK, P38, ERK) signal pathways can also be observed in PCH models and could result in transforming PCH to malignant heart failure [36]. Natural alkaloids berberine was proven to inhibit PCH through the AMPK pathway [21]. The CaN-NFAT signal pathway is regulated by Ca^2+^ and is directly related to the expression of fetal genes. Thus, this study focused on the CaN-NFAT signal pathway. CaN is a serine/threonine protein phosphatase. After activation by the upstream Ca^2+^-calmodulin complex, CaN dephosphorylates the p-NFAT protein and then promotes the nuclear localization of NFAT [37]. After being combined with GATA-4, the NFAT/GATA-4 complex stimulates hypertrophy-related fetal gene expression [38,39]. The NFAT protein family includes NFAT1 (NFATc2), NFAT2 (NFATc1), NFAT3 (NFATc4), and NFAT4 (NFATc3). The NFATc3 protein is highly related to vascular system development and is a major research object in PCH-related experiments [40]. The results demonstrated that ANGII induced a significant increase in CaN expression and decreased the phosphorylation of NFATc3. TF3 decreased the CaN protein level and enhanced p-NFATc3 protein expression, which indicated that TF3 inhibited the transmission of the hypertrophy signal at least partly through the CaN-NFAT signal pathway.

To unravel the inhibition mechanism of TF3 on the CaN-NFAT pathway, Ca^2+^ level determination and molecular docking were conducted. The Fluo-4 fluorescence intensity results indicated that TF3 could decrease intracellular Ca^2+^ levels. Ca^2+^ is a common signal transduction substance in cells. Intracellular Ca^2+^ is discharged from the endoplasmic reticulum. The lack of Ca^2+^ in the endoplasmic reticulum can activate the Ca^2+^ channel on the cytoplasmic membrane, resulting in the influx of extracellular Ca^2+^. Molecular docking is an important method in molecular simulation. Its essence is the recognition process between two or more molecules, which involves spatial and energy matching between molecules. In this trial, semi-flexible docking was used to measure the ability of TF3 with proteins. The results showed that TF3 could dock with calmodulin and CaN. Their binding energy was −3.76 kcal/mol and −0.5 kcal/mol, respectively (Figures S2 and S3). There were hydrogen bonds between TF3 and multiple residues of these two proteins. CaN is composed of an α subunit (catalytic subunits) and β subunit (regulatory subunit). TF3 could bind with two submits, which is different from an inhibitor of CaN, FK506 (only bind with α subunit) [41]. In all, TF3 could not only regulate the upstream Ca^2+^ level but also bind with calmodulin and CaN to down-regulate the CaN level, enhance p-NFAT expression, and eventually inhibit the activation of the CaN-NFAT signal pathway in PCH cells.

## 5. Conclusions

**Figure 7:**
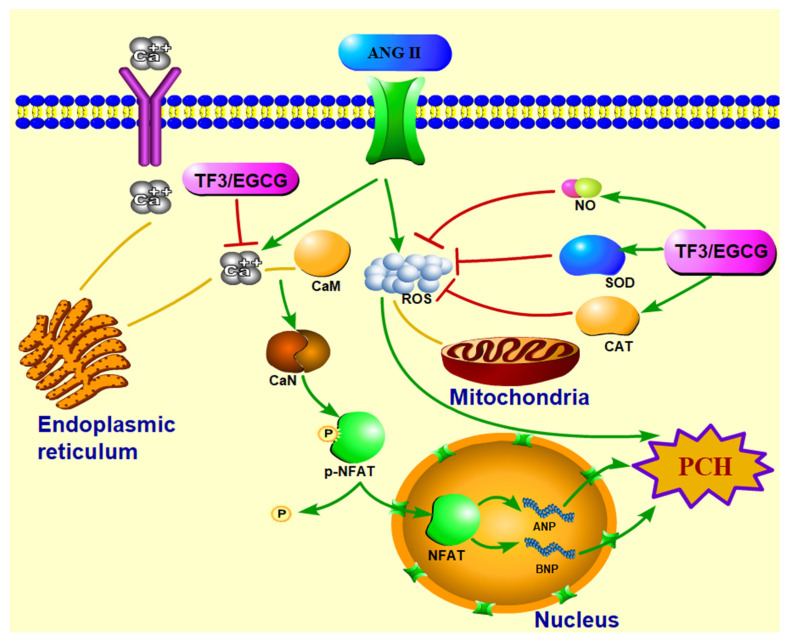
Diagrammatic sketch of TF3 on preventing H9c2 cells pathological cardiac hypertrophy induced by angiotensin II via CaN-NFAT signal pathway.

TF3 could significantly reduce the ANP and BNP mRNA expression and cell size in PCH cells induced by ANGII. Meanwhile, TF3 could maintain the redox balance during PCH formation. TF3 inhibited the activation of the CaN-NFAT signal pathway by decreasing the intracellular Ca^2+^ level, down-regulating the CaN level, and enhancing p-NFAT expression (Figure 7).

## Figures and Tables

**Table 1:** Sequence of primers for real-time qPCR.

Gene	Primer Sequences (5′-3′)	Annealing Temperature (°C)
ANPc ^1^	Forward: AAAGCAAACTGAGGGCTCTGCTCG Reverse: TTCGGTACCGGAAGCTGTTGCA	65.66 64.69
BNP	Forward: GTCAGTCGCTTGGGCTGT Reverse: CCAGAGCTGGGGAAAGAAG	59.97 57.43
GAPDH	Forward: CTCATGACCACAGTCCATGC Reverse: TTCAGCTCTGGGATGACCTT	58.62 58.34

^1^ ANP: Natriuretic peptide A, BNP: Natriuretic peptide B, GAPDH: Glyceraldehyde-3-phosphate dehydrogenase.

## Data Availability

Not applicable.

## Supplementary Materials

The following supporting information can be downloaded at: https://www.mdpi.com/article/10.3390/nu14071391/s1, Figure S1: Viability of H9c2 cells treated with different concentrations of EGCG, Figure S2: Molecular docking result of TF3 and calmodulin, Figure S3: Molecular docking result of TF3 and CaN.
