# Supplementary material for: The Prevention Role of Theaflavin-3,3′-digallate in Angiotensin II Induced Pathological Cardiac Hypertrophy via CaN-NFAT Signal Pathway
Source: Nutrients. 2022 Mar 26;14(7):1391. doi: 10.3390/nu14071391 (PMC9003418; doi:10.3390/nu14071391)
Supplement: Supplementary file 1 [file nutrients-14-01391-s001.zip › nutrients-1623303-supplementary.pdf]

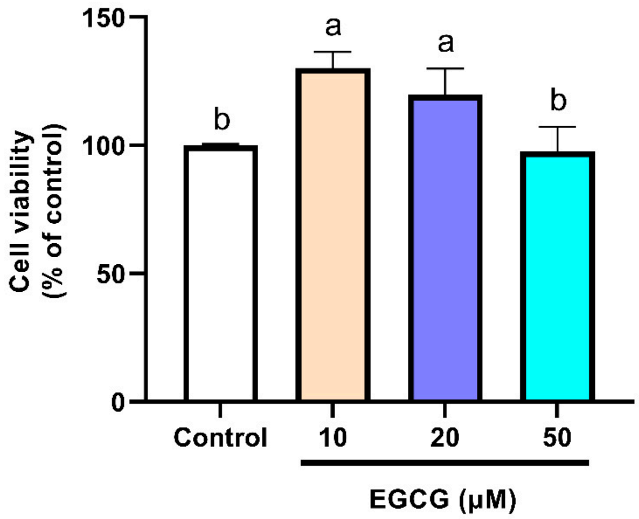

Figure S1. Viability of H9c2 cells treated with different concentrations of EGCG. Significant differences between different treatments were showed by different letters ($p < 0.05$).

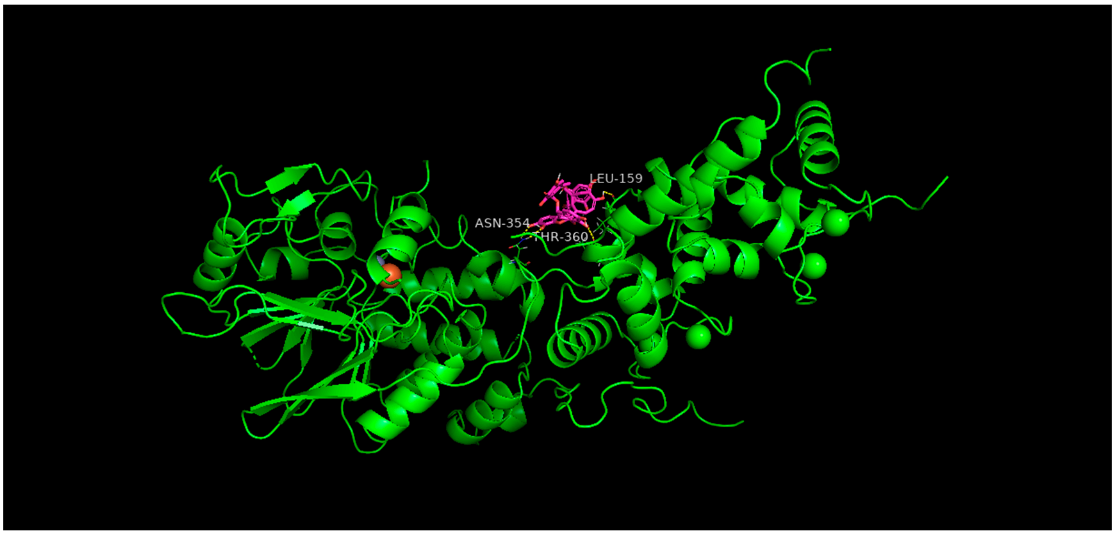

Figure S2. Molecular docking result of TF3 and calmodulin.

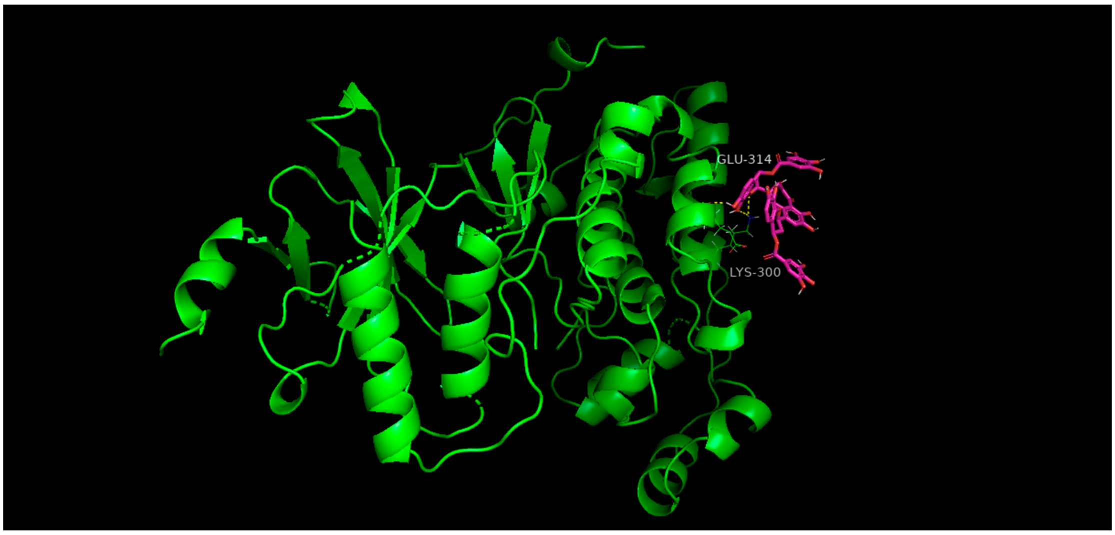

Figure S3. Molecular docking result of TF3 and CaN.
